# Food and Consumers’ Environment Inside and around Federal Public Schools in Bahia, Brazil

**DOI:** 10.3390/nu16020201

**Published:** 2024-01-08

**Authors:** Fabiana Chagas Oliveira de França, Renata Puppin Zandonadi, Ívenes Ariele da Silva Santana, Izabel Cristina Rodrigues da Silva, Rita de Cassia Coelho de Almeida Akutsu

**Affiliations:** 1 Nutrition School, Federal University of Bahia-Augusto Viana, s/n-Palácio da Reitoria, Canela, Salvador 40110-907, Brazil; fabianacofranca@hotmail.com (F.C.O.d.F.); contatoarielesantana@gmail.com (Í.A.d.S.S.); 2 Department of Nutrition, University of Brasilia, Brasilia 70910-900, Brazil; renatapz@unb.br; 3 Faculty of Ceilândia, University of Brasília, Brasília 72220-275, Brazil; belbiomedica@gmail.com

**Keywords:** food environment, consumer environment, ESAO-R, HMRI, school canteen

## Abstract

The food environment plays a crucial role in shaping people’s eating habits and, in and around schools, this influence becomes even more critical due to the time students spend daily in these spaces. This study aimed to analyze the food and consumer environments inside and around federal institutes in Bahia, Brazil. Ecological study and audit methodologies were combined, with all the 35 federal institutes in Bahia as the sample universe. To delimit the food environment around the school, the establishments were mapped in a 1000 m buffer, with the school as the centroid. The geographic coordinates of schools and food outlets were initially obtained using Google Maps and later confirmed on-site. The data were collected in WGS 84 and converted to UTM zone 23S SIRGAS 2000. To map the consumer environment, establishments found in a 250 m buffer and also canteens within schools were audited, using the ESAO-r instrument that considers the availability and prices of healthy and unhealthy foods; availability of nutritional information near the point of purchase or on the menu; and presence of internal marketing of healthy and unhealthy foods. The healthy meal–restaurant index (HMRI) was also measured. This index ranges from 0 to 8 points and positively scores items related to healthy consumption and fails to score those related to unhealthy consumption and behavior. The establishments were grouped into four categories (healthy, unhealthy, mixed, and supermarkets). The surrounding area with four or more unhealthy establishments within the 250 m buffer was considered a food swamp. Descriptive analyses were carried out with frequency measurements, measures of central tendency (mean and median), and dispersion (standard deviation). Around the analyzed schools, 732 food establishments were identified, 73.8% (*n* = 540) formal and 26.2% (*n* = 192) informal. Considering the characteristics of existing commerce, there was a predominance of snack bars (45%), grocery stores (23%), and bars (7.8%), with a smaller number of supermarkets identified (4.1%). School canteens were found in 20 schools analyzed and only 15% had a variety of healthy foods. When evaluating the availability of healthy foods, a median HMRI of 3 (1–7) was observed. When analyzing this index according to the type of establishment, it was found that fruit and vegetables presented higher values (HMRI = 7; P25–P75: 4–8) compared to large chain supermarkets (HMRI = 5; P25–P75: 2–6; *p* < 0.001) and local markets (HMRI = 4; P25–P75: 2–5; *p* < 0.001). From the analysis of the food and consumer environments, it was possible to make inferences about the quality of the food offered to students in these locations, as well as the potential health outcomes arising from this exposure and the need to carry out food education activities and nutritional activities.

## 1. Introduction

The food environment is a concept that encompasses individual factors (food microenvironment), such as the place where a person lives, works, and studies, and broader factors (food macroenvironment), such as public policies, food industries, and marketing strategies [1]. These two levels of influence, the food microenvironment and macroenvironment, play a crucial role in shaping people’s eating habits [2,3].

The microenvironment, which contemplates inside and around schools the existence of school policies regulating the food sale in canteens, promoting nutritional education, encouraging the offer of healthy foods, and limiting the availability of unhealthy products, can have a significant impact in improving children and adolescents’ diet [4,5,6]. In this context, creating a school environment that promotes the appreciation of a balanced diet and awareness of the importance of healthy food choices can contribute to an environment more conducive to health [5,6,7,8].

In Brazil, some studies have been performed to understand the food environment and interpret its influence on individuals’ quality of life and health outcomes derived from food choices. Peres et al. (2021), in a study carried out in Belo Horizonte, Brazil, identified a cluster of cafeterias, restaurants, and bars around the schools analyzed [2,3], as did Henriques et al. (2021), who identified that 15% of the daily energy consumed by students in Rio de Janeiro, Brazil, came from ultra-processed foods eaten around schools [5]. Comparing the regions of Brazil and the food environment, do Carmo et al. (2018) present data that the food environment in the northeast region of Brazil (where the sample of this study is concentrated) is more obesogenic than the other regions of the country [7]. A study carried out in Salvador, Brazil, a city that is part of the sample of this work, contextualizes the importance of food for students, mainly because they are present at school for a long time during the day, showing that the options sold at a more affordable price are those most consumed, despite not being the healthiest [9].

Taking advantage of this context and the need for daily nutrition, it is possible to analyze the consumer’s food environment, that is, the availability of healthy options in the places where people usually shop for foods, and its impact on diet and obesity. This approach considers not only the presence of any establishment but also the quality and accessibility of healthy food options available to consumers in their daily routines. Glanz et al. (2023) highlight the importance of a comprehensive understanding of the food environment and its complex interactions with health issues, emphasizing the need to consider both the community environment and the consumer environment when addressing issues related to diet and obesity [10].

It is, therefore, necessary to evaluate the relevance of strategies that encourage healthy food choices, as well as to recognize and overcome marketing influences and barriers that can induce consumers to choose less healthy foods. These actions can and should be carried out in Brazilian public schools as part of the implementation of the Brazilian National School Feeding Program (Programa Nacional de Alimentação Escolar—PNAE) [11], which determines the supply of fruits, vegetables, high-biological-value proteins, and grains, in addition to the application of guidelines for the promotion of healthy eating in kindergarten, elementary, and secondary schools of the public and private networks, instituted by national legislation (*Portaria interministerial* nº 1010/2006 [12]), through the implementation of healthy canteens. These modifications were made following the guidelines of the Brazilian Ministry of Health [13] and Law nº 13.666/2018 [14], which determines the inclusion of the food and nutrition education (FNE) in the scholar curriculum. In the macro-environmental scope, it is essential to consider government health and nutrition policies, food industry marketing strategies, and food labeling regulations, as such measures can indirectly influence scholars’ food choices [3,4,8].

The surroundings of schools are often identified as suitable spaces for the sale of food aimed at children and adolescents, such as soft drinks, snacks, sugary drinks, sweets, and candies [1,2,7,15]. It is important to recognize that the availability of these foods close to schools can negatively influence students’ food choices, especially adolescents, who generally have the autonomy to purchase and have the possibility of choosing the foods they will consume [2,6,16]. These products can represent an obstacle to the promotion of healthy eating habits, even in schools that have established feeding programs or opted to offer healthier options in their canteens [8,17].

The prevalence of overweight, obesity, and severe obesity statuses in the adolescent population is concerning. As indicated by data from the Brazilian Food and Nutrition Surveillance System (SISVAN), in the period from January to October 2022, 17.6% of adolescents aged between 10 and 19 years who were served by the Unified Health System (SUS) in the state of Bahia were overweight. Furthermore, 8.1% were diagnosed with obesity, while 1.82% were identified with severe obesity [13]. These data demonstrate the need to study the environment surrounding these individuals better and establish control mechanisms to improve health and quality of life.

Given the context presented, we consider that there is evidence that some food establishments, especially those selling unhealthy foods, are concentrated around schools and that adolescents have the autonomy to buy food inside canteens and nearby commercial food establishments. In addition, no studies have explored the analysis of the food environment around federal public schools in the state of Bahia, Brazil, which denotes the need for investigation. This study aimed to analyze the food environment inside and around the federal public schools in Bahia, Brazil.

## 2. Materials and Methods

### 2.1. Study Design and Sample Characterization

This study combined ecological and audit study methodologies. Ecological studies map the food environment based on secondary data from the registration of establishments that sell food, with an observation regarding the qualitative aspects of the food served in these environments (restaurants, cafeterias, canteens, grocery stores). Audit studies, in turn, aim to collect information about the quality and availability of food and consumer products inside establishments [1]. In Brazil, public schools are divided into municipal, state, and federal schools. The present study was performed in all 35 federal public schools of Bahia, Brazil, and data were collected from November 2022 to May 2023. Municipal data for estimated population, estimated income, gross domestic product (GDP), and human development index (HDI) were obtained from public data available at the Brazilian Institute of Geography and Statistics (Instituto Brasileiro de Geografia e Estatística—IBGE) [18]. Data on vulnerability were obtained through the technical document containing the update of territories with the highest levels of Food and Nutrition Insecurity in Brazil [19]. The study was approved by the Federal University of Bahia Research Ethics Committee under registration CAAE: 57747322.5.0000.5023, 11 April 2022.

### 2.2. Data Geocoding

The geographic coordinates (latitude and longitude) of schools and food outlets were initially obtained using the online search service Google Maps (https://www.google.com.br/maps?hl=pt-BR, accessed on 5 November 2022) and later by an active search around the schools, with real marking of the location obtained. This methodology has been successfully replicated by other authors [1], having been adopted for this reason. The data were collected in the WGS 84 Geographical Coordinate System configuration and subsequently transformed to the Projected Coordinate System, Universal Transverse Mercator System (UTM), 23S time zone, SIRGAS 2000 datum, using the software QGIS version 3.30.2 (Brazil, 2021).

### 2.3. Characterization of Food Environment around Schools

To delimit the food environment around the schools, this study considered all food commercial establishments (formal and informal) located within a radius of 1000 m, with the school as the center of the analyzed buffer. Formal establishments were those registered with the Municipal Finance Department, and informal establishments did not have registration. This distance was selected considering a systematic review of the literature, carried out by França et al. (2022) [1], which revealed that this route can be covered on foot in a maximum time of 20 min by adolescents who are students of the analyzed schools, and which involves food establishments that students can have close contact with when purchasing snacks daily and still commuting to and from school.

From the literature review on the products sold, their processing degree, and the association between the type of food establishment and consumption [1], we classified food establishments into four categories (healthy, unhealthy, mixed, and supermarkets). Healthy food establishments were those that sold mainly in natura or minimally processed foods (grocery stores; butchers and fishmongers; natural product stores; healthy food street vendors and fairs); unhealthy food establishments were those that sold predominantly ultra-processed products (convenience stores; bars; bombonier; beverage distributors; snack bars; ice cream parlors; and unhealthy food street vendors); mixed food establishments were characterized by the sale of healthy and unhealthy foods (bakeries and restaurants); and supermarkets were placed in a separate category because they sell a wide variety of foods and because there is no consensus in the literature on the influence of these establishments on individuals’ food consumption.

We emphasize that to arrive at this methodology, a systematic scoping review of the literature was previously carried out by França et al. (2022), which demonstrated how ecological and auditing studies have been carried out around the world, how establishments are characterized, and which distances are used to better characterize the food environment around schools, especially for teenagers [1].

### 2.4. Characterization of Food Swamps around Schools

To identify food swamps around schools, an adaptation of the methodology proposed by Hager et al. [20] was used, which considered neighborhoods with a high availability of convenience stores and small grocery stores as food swamps, and in this study, we included cafeterias, as they are an environment frequented by teenagers and are associated with unhealthy eating patterns. For the calculation, the number of snack bars, grocery stores, cafeterias, fast food restaurants, and candy stores around the schools located within a radius of 250 m, with the school as the center of the analyzed buffer, was added and when a total greater than or equal to four establishments was found, the location was classified as a food swamp.

### 2.5. Characterization of Consumers’ Food Environment around and Inside Schools

For food establishments located within a radius of 1000 m, with the school as the center of the analyzed buffer, an audit was performed, considering the possibility of purchasing food from the surroundings for consumption during school break time, in which students buy and eat the food and return to class, lasting from 20 to 30 min.

Data collection occurred between November 2022 and May 2023, through the application of an adaptation of the audit instrument for establishments selling food for immediate consumption, the ESAO Restaurant Observation Tool (ESAO-R) [21], a methodology validated nationally and that works with the assessment of the obesogenic food environment through direct observation of the site.

The ESAO-R included availability and pricing of healthy and unhealthy foods; availability of nutrition information near the point of purchase or on the menu; and presence of indoor food marketing (signs, table tents, or other displays that highlight healthy options and/or energy-dense foods (cookies, sugar-sweetened beverages, fries, burgers) on the menu).

The quality and cost of food were assessed considering the items most reported by adolescents in the Brazilian Research of Family Budgets 2017–2018 [22]. We measured the cheapest variety found of a given fruit or vegetable and the cost was based on the posted prices per kilogram. When only price per unit was available, we weighed three random units, averaged their values, and calculated the price per kilogram. Variety was measured as the number of different types of fruits and vegetables within each kind. We measured the variety for selected ultra-processed foods by the number of different brands available for purchase. The variety of soda, other sugar-sweetened beverages, snacks, and cookies were measured by the number of different brands and flavors.

Marketing was measured by counting different signs or advertisements that promoted the purchase of healthy or unhealthy foods, such as signs with nutrition information, signs or other displays that encourage purchasing or eating such products, and discounts.

Raters (undergraduate and graduate students) assessed all food stores, open-air food markets, and restaurants within the sampled buffer. Their training consisted of four sections of two-hour instruction, followed by two to three hours of practice, and ESAO’s application manuals were used for this training.

In addition to the analysis of the school surroundings, an audit of the school canteens was performed, through the analysis of the availability and types of food sold, with completion of the audit checklist ESAO-R to add the high- and low-nutritional-quality diet markers available in the Brazilian Research of Family Budgets 2017–2018 [22].

Based on data collected through ESAO-r, the healthy meal–restaurant index (HMRI) can measure access to healthy foods. This index ranges from 0 to 8 points and positively scores items related to healthy consumption and fails to score those related to unhealthy consumption and behavior. Items referring to unhealthy items and barriers to healthy eating were negatively coded and variables and scores are described in Table 1.

### 2.6. Statistical Analysis

The data were inserted in a single spreadsheet and then grouped according to the analysis required for each proposed methodology. Descriptive analyses were carried out with frequency measurements, measures of central tendency (mean and median), and dispersion (standard deviation). For this purpose, the statistical software SPSS 28.0.1 was used.

## 3. Results

Of the 35 schools analyzed, 54.3% (*n* = 19) are in the urban area, while 45.7% (*n* = 16) are in the rural area. In addition, considering the distance between the school and the city center, an average of 4.7 km and a median of 3.9 km were found, ranging from 0.5 km to 17 km. Around the analyzed schools, 732 food establishments were identified (Table 2). Of them, 73.8% (*n* = 540) were formal establishments and 26.2% (*n* = 192) were informal. Considering the characteristics of existing trade, there was a predominance of snack bars (45%), grocery stores (23%), and bars (7.8%), with a smaller number of supermarkets identified (4.1%).

Regarding the location of schools, 8.6% (*n* = 3) are in small cities (population less than 20 thousand inhabitants); 42.8% (*n* = 15) are in medium-sized cities (population between 20 thousand and 100 thousand inhabitants); and 48.6% (*n* = 17) are in large cities (population greater than 100 thousand inhabitants), according to data estimated by IBGE in 2021 [18].

In healthy establishments, the median number of units differed according to the type of school (higher median in IFBA) and the size of the municipality (higher median in larger municipalities); on the other hand, in unhealthy establishments, mixed establishments, and supermarkets, there was a statistical difference in all variables, and in unhealthy establishments and supermarkets, all the medians were different from zero.

In this context, we analyzed the concentration of food establishments located within a radius of 250 m, according to the methodology used, with the school as the center of the analyzed buffer, and found 180 places (Table 3) classified as healthy, unhealthy, mixed, and supermarkets. Those predominantly selling unhealthy foods were present in 57.2% (*n* = 103), followed by mixed, 31.7% (*n* = 57); supermarkets, 7.8% (*n* = 14); and healthy, 3.3% (*n* = 6). In addition to the analysis of the school surroundings, an audit of the school canteens was carried out, through the analysis of the availability and types of food sold, with completion of the audit checklist adapted to add the high- and low-nutritional-quality diet markers available in the Brazilian Research of Family Budgets 2017–2018 [22].

School canteens were present in 57.1% (*n* = 20) of schools analyzed. Informal street commerce immediately available in front of schools was also identified in 54.3% (*n* = 19) of schools.

**Figure 1:**
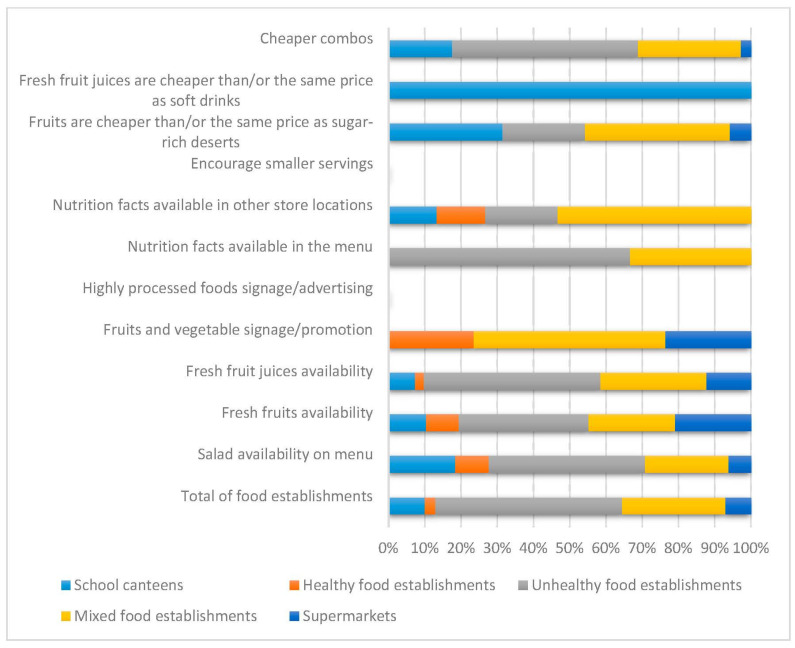
Assessment of the percentage contribution of food environment variables concerning the items evaluated using the ESAO-R tool.

The consumer’s food environment concerns how food is presented in places of purchase and consumption. This includes its packaging, information on the packaging, format, size, storage, preparation, cost, and nutritional quality. Based on these aspects, establishments that sold food within a buffer of 250 m from schools were evaluated using the ESAO-r tool and the analysis data are presented in Figure 1. The lack of an incentive to purchase smaller portions draws attention to food, as well as the absence of warning signs regarding processed and ultra-processed foods, which were not found anywhere.

Based on data collected using the ESAO-r tool, access to healthy foods can be measured using the Healthy Meals Index (HMRI). This index varies from 0 to 8 points and positively scores items related to healthy consumption and does not score those related to unhealthy consumption and behavior. When evaluating the availability of healthy foods, a median HMRI of 3 was observed in the establishments investigated, ranging from 1 to 7. When analyzing this index according to the type of establishment, it was found that fruit and vegetables presented higher values (HMRI = 7; P25–P75: 4–8) compared to large chain supermarkets (HMRI = 5; P25–P75: 2–6; *p* < 0.001) and to local markets (HMRI = 4; P25–P75: 2–5; *p* < 0.001). These data lead us to conclude that fruit and vegetables can provide better access to the population concerning large supermarket chains, due to their marketing profile of the majority of fresh or minimally processed products.

## 4. Discussion

### 4.1. School Food Environment and Food Swamps around Federal Schools

The concentration of food establishments in the central regions of cities is due to the greater circulation of people and the buying and selling relationship established in these places. A study by Leite et al. (2021) in Minas Gerais, Brazil, identified a concentration of food establishments around schools that tend to be concentrated close to the busiest neighborhoods [15]. In addition, the authors observed that the density of food trade around the schools decreased as the local vulnerability increased, suggesting a relationship between the location of the schools, the availability of food, and the socioeconomic situation of the residents of those places.

Concerning the location in the rural area, there is an intrinsic difficulty in maintaining commercial activities in the food sector, whether due to the lack of concentration of consumers in those locations, the logistical difficulty of transport, storage, and trade, or even the scarce security for the customers [10,23]. A study by Joyce et al. (2020) in the USA demonstrated that when related to socioeconomic status, location in a rural area plays a significant role in many health disparities, including issues related to nutrition [23]. In studies carried out in Brazil, there were no parameters for comparison, specifically between urban and rural areas, since they did not present these data [2,15,16].

Studies previously carried out in Brazil considered large cities to analyze the food environment around schools [2,15,16,24,25]. This work confirms what was previously stated, in the sense of the concentration of establishments around schools, as it was greater in large municipalities, sixteen times and three times greater, compared to small- and medium-sized ones, respectively.

Among the 16 schools located in the rural area, 19% (*n* = 3) are at a distance greater than 10 km from the urban center. There were only two food establishments within a 1km radius of these schools, including the canteens. In these places, food alternatives are lacking for students who need to be fully assisted by existing food programs. As this is a public primary education school, it is even more critical to fully implement the PNAE [11] in these schools to guarantee the right to adequate and healthy food for students and to meet their daily nutritional needs following the assumptions of the current legislation [8,11,26].

In Brazil, previous studies have shown that cafeterias are predominant near schools, both in kindergarten and elementary school and high school, regardless of whether public or private [2,5,16,25]. The literature on the food intake preferences of adolescent students is well established and shows essential data regarding the excessive consumption of unhealthy foods, including sugary drinks and foods low in fiber and nutrients, by this part of the public, as well as the direct relationship between eating behavior, excess adiposity, and risk of coronary diseases in this group [7,27,28,29]. Given this situation, according to the data obtained in this study, the presence of cafeterias and grocery stores around schools can favor the acquisition of unhealthy foods sold in these places, which can have negative consequences for students’ health.

Despite the Brazilian legislation prohibiting the sale of alcoholic beverages to persons under 18 years of age [30], the presence of bars around schools was also evidenced in other studies [2,15,16] and is highlighted since, according to the 2017–2018 Brazilian Household Budget Survey [22], beer (65.4%) and wine (49.9%) are the items with the highest percentage of out-of-home consumption among adolescents, often being related to a phase of rebellion, self-affirmation, and contestation of imposed rules [27,28]. These consumption data are worrying since alcohol consumption is associated with risks to the health and well-being of this age group, such as impaired cognitive and behavioral development; mental health issues such as depression and anxiety; school performance and relationships with peers and family; traffic accidents, injuries, and falls; addiction and dependency; risky sexual behavior; physical damage such as liver disease, pancreatitis, and peripheral neuropathy [31,32,33]. In this context, joint work between family, school, and government must promote health education to make adolescents aware of the risks of alcohol consumption, as well as strengthen the inspection of the sale of alcoholic beverages to minors [31,32,33].

There is at least one unhealthy establishment within the 1000 m buffer surrounding all federal schools participating in this study. Concerning the proximity of food-selling establishments, the distance from a federal school to the nearest unhealthy establishment was almost five times shorter when compared to a healthy establishment. This demonstrates that in this study, federal schools are closer to places that sell unhealthy food, confirming the data already presented in previous studies [3,7,16,24].

Several studies have shown that this large concentration occurs to attract consumers who have an affinity to the type of food sold and that it is rare to find healthy establishments around schools in large urban centers [2,16,34]. In addition, studies on food swamps are growing, which refer to saturated environments of establishments where the sale of high-calorie products with few nutrients predominates, as in the case of fast food chains and convenience stores [2,34].

In this study, 40% (*n* = 14) of schools were located in regions classified as food swamps, according to the methodology proposed by Hager et al. [20]. A study carried out by Peres et al. (2021), which analyzed all public and private schools (*n* = 1436) in Belo Horizonte, Brazil, found that 54.6% of schools were located in food swamps, with the highest density around private high schools and higher-income census tracts [2]. Andretti et al. (2023), in a study carried out in Rio de Janeiro, Brazil, considering 3159 schools, identified the presence of food swamps in 97% of the neighborhoods analyzed, and they are also more prevalent around private high schools and in the highest-income tertiles [35]. Also, regarding food swamps, one can notice the disparity between the location of the school in the urban area and the population’s income concentration.

The impact of food swamps on adolescents’ dietary choices is a crucial factor that cannot be overlooked, given its strong association with adverse health consequences. In Brazil, the prevalence of ultra-processed food sales in cafeterias and bars is significant [36]. Research conducted among Brazilian children and adolescents has revealed a positive correlation between patronizing such establishments and an increased consumption of ultra-processed foods [16,27,37,38]. Furthermore, investigations examining the food landscape surrounding schools in various countries have consistently shown that the available food options generally lack nutritional quality, thereby subjecting children and adolescents to an obesogenic environment [37,38].

The literature discusses the obesogenic environment caused by the excessive availability of foods of low nutritional value. Data from Do Carmo et al. (2018) about the food environment around Brazilian schools showed that the food environment in the Brazilian northeast region stood out as more obesogenic than the other regions of Brazil, with predominance in the surroundings of private schools [7]. Henriques et al. (2021) analyzed and compared the types of food sold in the surroundings of schools in Rio de Janeiro, Brazil, and found similar data. The authors reported that hot dogs, sandwiches, and snacks (fried and roasted) had a percentage above 15% of energy contribution from ultra-processed foods [5], contrary to PNAE recommendations for this age group [11].

A study in New Zealand showed that the food environment around schools, even with a small effect, can influence adolescents’ food quality [6]. Evidence also suggests that the food environment around schools can affect adolescents who are ethnic minorities, belong to low-income classes, and live in urban areas more negatively since their exposure to the obesogenic food environment is greater, in addition to having higher rates of excess weight and associated chronic diseases [4]. In contrast, a cohort study carried out in Brazil followed the diet of adolescents from public schools for three years and showed a higher daily frequency of consumption of ultra-processed foods at the beginning of the study with a decrease over time, but an increase in weight and body mass index (BMI), suggesting that there is a greater relationship with the quantity than with the quality of food ingested [39]. Thus, more studies are needed to monitor the changes in food intake and body composition of adolescents so that more assertive results can be generated about this correlation.

### 4.2. Consumers’ Food Environment around and Inside Federal Schools

Due to the time spent on the journey and having a snack within the school break time, adolescents likely concentrate their purchases of food in establishments located in the immediate surroundings of schools and, in the audit carried out in these places, the main foods consumed by adolescents can be configured as markers of high- or low-nutritional-quality diets. A low offer of healthy options for students, especially natural juices and fresh fruit, was available in less than 50% of school canteens, cafeterias, and bakeries. In contrast, soft drinks, sugary drinks, snacks, and sweets were present in all establishments. Similar results were found in studies that evaluated the food environment around Brazilian schools [2,3,15,16,17,24,25], reflecting on the consumption pattern characteristic of the age group and the need for implementation policies to promote healthy eating for teenagers.

Although there is a guiding manual on promoting healthy habits in school canteens [13], Brazil lacks specific federal legislation regulating the sale of food in school canteens. However, several Brazilian states and municipalities have taken the initiative to create their legislation [40,41,42,43,44,45] to regulate this issue, seeking to promote the offer of healthier foods to students and combat childhood excess weight. These state and municipal laws vary in terms of requirements and restrictions for food sold in school canteens but, in general, they establish guidelines for offering more nutritious foods and restricting products with a high sugar content, saturated fats, and sodium. There are no specific laws for the state of Bahia, where this study was performed.

Due to the commonly more affordable prices and ease of access, street commerce, also called “street food”, is the first choice of adolescents, and the products sold in these places were mostly markers of a low-nutritional-quality diet. “Street food” represents a significant part of a region’s food culture, often offering traditional dishes and local specialties, rich in sugars and fats, having raised discussions about food and nutritional security involved in the process [46]. In a study comparing the regions of Brazil, the sale of “street food” at the entrance gate and around the schools was around 60% in the northeast region and was characterized by the predominant sale of foods of low nutritional value, such as soft drinks, sugary drinks, snacks, sweets, and treats [7].

Data presented by the 2017–2018 Brazilian Family Budget Survey show that per capita consumption of low-nutritional-quality diet markers was higher among adolescents, notably for stuffed biscuits, whose average consumption was almost four times higher among adolescents (9.7 g/day) than in adults (2.5 g/day) and 16 times higher than that of older people (0.6 g/day); soft drinks, for which the average consumption was 3.7 times higher among adolescents than among older people; chips, whose average consumption among adolescents was four times higher than that of adults and 20 times that estimated for older people; sandwiches, for which the average consumption of adolescents was twice that observed for older people; and pizzas, whose average estimated consumption for adolescents was four times that of older people [22].

Glanz et al. (2023) conducted a systematic literature review that revealed a notable pattern in research regarding food environments. According to the authors, most studies address the community food environment, highlighting elements such as the presence, density, and type or category of commercial food establishments, and a small number of studies are dedicated to exploring the connections between the consumer’s food environments. The authors conclude from this fact that there is a smaller availability of healthy options in the places where people usually shop for food, which impacts diet and obesity [10].

When discussing the consumer’s food environment, the implementation of specific strategies emerges as a catalyst for promoting healthier food choices. Studies show that the supply and diversity of healthy food products at promotional prices, as well as the prominent positioning of fruits and vegetables at the store entrance and in strategic locations, are associated with increased purchases by consumers [10]. On the other hand, the literature also identifies several frequent barriers that influence purchasing behavior, often leading the consumer to purchase unplanned and unhealthy products in most cases, and two groups can be considered relevant, namely marketing and spatial configuration of establishments. Marketing, advertising, tasting, and promotions associated with ultra-processed foods have been strongly associated with the increase in the acquisition of these foods, even if they were not planned [47].

Access to healthy foods is influenced by the characteristics of the food environment, especially the consumer’s food environment, which includes attributes related to the availability, variety, quality, price, and promotion of food products in commercial establishments. Understanding the relationships between the consumer’s food environment and the dynamics of food purchasing by students can contribute to elucidating the factors that impact individuals’ eating patterns.

A study carried out in Barcelona, Spain, based on the analysis of food establishments in a buffer of 400 m around public and private schools, found 95% (*n* = 146) of establishments classified as unhealthy. In total, 90% of schools had at least two unhealthy establishments nearby. The authors reported a positive association between schools located in higher-income neighborhoods and greater availability and accessibility of healthy food products, with strong social inequalities found in the supply of healthy foods [48].

Glanz et al. report that, in an analysis of the available literature, differences in access to healthy foods, driven both by the location of establishments and the availability of products, can create disparities in food environments with possible health implications, which, by favoring those who live in wealthier areas, may ultimately contribute to widening health inequalities [10].

### 4.3. Study Limitations

Despite having established a buffer of 1000 m from the center of the school, this measurement does not consider the routes that are used to access schools, and it is possible that students do not have real access to these places. Despite this limitation, this strategy has been widely used in studies that evaluate the food environment in the school environment. Verification of the food establishments was carried out over a period of just two days in each city, which may have caused an undersizing of street commerce around the schools.

Furthermore, some evaluations that were not possible in this work should be considered for future studies, such as evaluating the student’s route; prices of food being marketed and its influence on the choice; and objective evaluation related to street food vendors.

## 5. Conclusions

It was found that the food environment around federal schools in Bahia has heterogeneous characteristics related to the size of the municipalities, the location of the school in an urban or rural area, and the distance from the school to the city center. It was possible to identify environments with little or no access to food around the school and others with a very high concentration, resembling food swamps. In both contexts, the importance of correctly implementing the National School Meal Program (PNAE) is evident to provide schoolchildren with quality meals that meet their daily nutritional needs. Furthermore, the need to carry out food and nutritional education activities and educational activities in partnership with schools, governments, and families was highlighted to avoid risky behaviors and diseases associated with low-nutritional-quality food.

Concerning the consumer environment, it was possible to identify most food markers of low-nutritional-quality diets, as well as high caloric value, composed of excess sugars and fats, which become a concern when it comes to the quality of life and health of adolescents, as well as increasing the likelihood of developing chronic diseases associated with overweight and obesity statuses.

The absence of legal provisions at the state and federal level that serves the municipalities involved was considered, requiring the implementation of more comprehensive regulations that address the sale of unhealthy foods in the vicinity of schools, as well as educational and awareness initiatives for students, parents, teachers, and the community at large about the importance of healthy food choices.

More studies are needed to expand knowledge about food environments, their influence on adolescents’ choices, the consequences of this environment on health and quality of life in adolescence, and future implications. Promoting an environment that favors the supply and choice of nutritious foods should be a management priority. Through it, it will be possible to contribute more effectively to promoting healthy eating, even when less healthy products are available near schools.

## Figures and Tables

**Table 1:** Scoring system for the Healthy Meal—Restaurant Index (HMRI).

Variable	Score
Salad bar availability	0 points if not available; 1 point if available
Fresh fruit availability	0 points if not available; 1 point if available
Fresh fruit juice availability	0 points if not available; 1 point if available
Fruit and vegetable signage/promotion	0 points if not available; 1 point if available
Highly processed food signage/advertising	0 points if available; 1 point if not available
All-you-can-eat buffet only	0 points if available; 1 point if not available
Nutrition facts available on the menu	0 points if not available; 1 point if available
Nutrition facts available at other store locations	0 points if not available; 1 point if available

**Table 2:** Classification of food establishments according to the type of school, size of the municipality, and location of the school, considering a 1000 m buffer.

		Healthy Establishments		*p*	Unhealthy Establishments		*p*	Mixed Establishments		*p*	Supermarkets		*p*
		Median	Range		Median	Range		Median	Range		Median	Range	
According to the type of school	IFBAIANO (*n* = 14)	0	2	0.056 *	1	21	0.026 *	1	11	0.002 *	1	6	0.020 *
	IFBA (*n* =21)	1	8		7	57		7	34		4	13	
According to the municipality size	Large (*n* = 17)	1	8	0.006 *	11	57	0.028 *	8	35	0.015 *	6	13	0.031 *
	Medium (*n* = 15)	0	2		3	20		3	18		2	9	
	Small (*n* = 3)	0	0		1	6		0	4		1	2	
According to the school location	Rural area (*n* = 19)	0	2	0.182	1	21	0.008 *	2	26	0.015 *	1	7	0.024 *
	Urban area (*n* = 16)	0	8		7	57		7	35		4	13	

* *p*-value < 0.0001, showing a significant difference in scores between location and type of school.

**Table 3:** Availability of foods/beverages considered markers of high and low nutritional quality by types of food establishments located inside or within 250 m of the schools.

	School Canteens *n* (%)	Healthy Establishments *n* (%)	Unhealthy Establishments *n* (Amplitude)	Mixed Establishments *n* (%)	Supermarkets *n* (%)
Total	20 (100%)	6 (100%)	103 (100%)	57 (100%)	14 (100%)
High-nutritional-quality diet markers					
Fresh juices ^1^	17 (85%)	6 (100%)	60 (58%)	17 (30%)	6 (43%)
Fresh fruits	12 (60%)	6 (100%)	62 (60%)	12 (21%)	14 (100%)
Healthy meals ^2^	20 (100%)	5 (83%)	45 (44%)	8 (14%)	11 (79%)
Low-nutritional-quality diet markers					
Sugary beverages	19 (95%)	0	103 (100%)	57 (100%)	14 (100%)
Stuffed cookies	17 (85%)	0	91 (88%)	42 (74%)	14 (100%)
Hot dog/hamburger	15 (75%)	0	82 (80%)	20 (35%)	3 (21%)
Candies	17 (85%)	0	103 (100%)	57 (100%)	14 (100%)
Fried snacks	19 (95%)	0	75 (73%)	30 (53%)	9 (64%)
Confectionery ^3^	20 (100%)	1 (17%)	72 (70%)	27 (47%)	7 (50%)
Soda	19 (95%)	2 (33%)	103 (100%)	51 (89%)	14 (100%)
Snacks	18 (90%)	0	98 (95%)	29 (51%)	14 (100%)

^1^ Coconut water, fresh natural juices, or juices prepared from frozen pulp; ^2^ couscous, tapioca, natural sandwich, or root-based snacks; ^3^ homemade cakes, pies, sweets.

## Data Availability

Data are contained within the article.
